# Annual Report of the National Board of Health, for 1879

**Published:** 1880-02

**Authors:** 


ANNUAL REPORT OF THE NATIONAL BOARD OF HEALTH, FOR 1879. Washington, D. C., January 1, 1880.

We have just received the *National Board of Health Bulletin*, with a supplement containing the regular annual report of the national health board, and we are sure the readers of the JOURNAL AND EXAMINER will be interested in knowing what the board has accomplished during the first year of its existence, and what it proposes to do in the future; hence we shall make copious extracts from the report. With rather questionable good taste, the board commences its report, not only with an account of its own organization, but also with copies of the cordial indorsements given to it by the American Public Health Association and the National Academy of Sciences. The past and future work of the board is then given as follows:

"II. The collecting of information with regard to the sanitary condition of some of the principal cities and towns in the United States. Work in this direction was organized at the first session of the board by the printing, with additions and corrections, of a series of schedules of questions for the sanitary survey of a city, the original schedules for this purpose having been prepared several years previously by a committee of the American Public Health Association. A number of these schedules have been returned to the board properly filled up, and others are in course of completion. It is believed that the result has already been good in indicating the lines of inquiry which should be pursued by municipal authorities in relation to sanitary matters, and that hereafter a large amount of valuable information will be collected by this means.

"III. The appointment of a commission to investigate yellow fever in the Island of Cuba. The commission, generally known as the Havana Yellow Fever Commission, consisting of Dr. S. E. Chaillé and Col. T. S. Hardie, C. E., of New Orleans; Dr. John Guiterás, of Philadelphia, and Surgeon George M. Sternberg, U. S. A., sailed for Havana on the 4th of July and returned on the 4th of October. The preliminary report of this commission has been printed as a supplement to the *National Board of Health Bulletin*, and a copy is herewith appended, marked B.

"From this report it appears that yellow fever must be considered as endemic in the Island of Cuba, and that for many years it has prevailed annually in the principal ports. The facts presented do not confirm the theory of the spontaneous origin of the yellow-fever poison on board ships, and make it improbable that the cleansing of the harbor of Havana and the constant renewal of its waters, however desirable, would prevent the infection of the shipping at this port. Attention is especially invited to that portion of the report which gives an account of the obstacles placed by the authorities of Cuba in the way of the execution of the law approved June 3, 1879, and to the action taken by the governor-general of Cuba in July, 1866, declaring foul all the ports of the United States, and decreeing that all vessels arriving from them at the ports of Cuba should be subjected to the most rigorous quarantine, in consequence of the report of cholera having appeared in Philadelphia. The recommendation of the report that an attempt should be made to establish an international sanitary code, and the reasons given therefor, also merit careful consideration. In this connection, however, it is to be remarked that, under the existing constitution of the United States, and the construction placed by judicial authorities as to the scope of the police powers of the several States, it may be impossible that the United States government should become a party to any international code of quarantine, properly so called, for the reason that it would be impossible to guarantee that, if such a code were agreed upon and a vessel coming from a foreign port had complied with all its requirements, such vessels could be admitted into any port of the United States, since it is within the power of any State, and of many municipalities, to impose a quarantine of delay, or even of non-intercourse, upon any vessel arriving at their port or ports. It is for this reason that the board has attempted to secure at present only an international system of notification as to the sanitary condition of ports and vessels.

"IV. The sanitary committee of the board on sanitary legislation, consisting of Drs. H. I. Bowditch, S. M. Bemiss and Stephen Smith, has employed a competent legal authority of Boston to collect and collate the sanitary laws of the United States and of the several States, including not only the statutes, but the decisions of the several courts, on all questions involving the public health. A copy of this work is herewith appended, marked C.

"This compilation is of such immediate practical interest and utility to all who are interested in sanitary legislation, that it is recommended that authority be at once granted for having it printed as a separate volume.

"It needs but a brief examination of this record to show that the sanitary legislation of the great majority of the States is in a very unsatisfactory condition. Even those which have provided State boards of health have not done so on any uniform or well-considered plan; and very few of such State boards are so organized as to be able to meet the emergencies of an epidemic.

"It is hoped that the publication of this compilation of sanitary law will be one means of securing efficiency, and, to a certain extent, uniformity, in both State and municipal sanitary legislation throughout the country.

"V. At the request of the board, an investigation as to the best method of determining the amount and character of organic matter in the air has been undertaken by Prof. Ira Remsen, of the Johns Hopkins University of Baltimore. A copy of the preliminary report of Prof. Remsen on this subject is herewith submitted, marked D.

"VI. An investigation as to the effects of disinfectants, and especially of those used for disinfecting inclosed spaces, has been undertaken by Dr. W. S. Bigelow, of Boston, assisted by Dr. H. P. Bowditch, professor of physiology, and Dr. Wood, professor of chemistry, of Harvard University, the whole being under the general direction and advice of Dr. C. F. Folsom, secretary State board of health of Massachusetts.

"VII. An investigation as to the composition and merits of the various patent disinfectants, which has been made at the request of the board, by Prof. C. F. Chandler, of Columbia College, president of the board of health of New York City.

"VIII. An investigation as to the prevalence of adulterations in food or drugs in the United States, under the direction of Dr. H. A. Johnson, chairman of the standing committee on this subject. In this connection a paper upon the deteriorations and adulterations of food has been prepared for the board by Dr. R. M. Kedzie, president of the State board of health of Michigan, and a paper on the adulterations of drugs, by Prof. L. Diehl, of Louisville, Ky. Copies of these reports are herewith appended, marked E and F.

"IX. A preliminary inquiry as to the diseases of food-producing animals of the United States and the legislation, whether State or national, which seems desirable in regard to this subject.

"This has been under the direction of a standing committee of the board composed of Drs. J. L. Cabell, T. S. Verdi and P. H. Bailhache, and special reports upon the subject for this committee have been prepared by Prof. James Law, of Cornell University, New York, by A. Laintard, veterenary surgeon, New York, and by T. S. Verdi, member of the board. Copies of these reports are herewith appended, marked G.

"X. An investigation of the flow of sewers in relation to their sizes and gradients.

"This work has been carried on under the direction of Col. George E. Waring, jr., of Newport R. I., with a view to determining the most efficient and least expensive form of sewers.

"The preliminary report of Col. Waring on this subject is herewith appended and marked H. The investigation is still in progress, and will be completed during the ensuing spring.

"It may be observed that the results of this investigation have already been of practical importance, since it was the presentation of a portion of these results to the committee charged with the sanitary survey of Memphis which led that committee to recommend a scheme for the sewerage of that city, based on the researches of Col. Waring, estimated to cost $225,000, in preference to a plan of sewerage prepared in the ordinary manner and estimated to cost $500,000.

"XI. A sanitary survey of the eastern coast of New Jersey, bordering on New York harbor is in progress, with the aid of this board, under the direction of the State board of health of New Jersey.

"XII. A sanitary survey of the city of Memphis, Tenn. This was commenced as soon after the close of the epidemic as possible, and is still going on, being under the direction of a special committee of the National Board of Health, of which Dr. J. S. Billings is chairman. A preliminary report of this committee, prepared to meet the urgent demand on the part of the municipal authorities for advice as to the course to be pursued is herewith submitted, marked K.

"XIII. An investigation as to the hygiene of the mercantile marine and as to what legislation is expedient to improve its sanitary condition. The report of Surgeon P. H. Bailhache, U. S. M. H. S., especially detailed by the chief of that bureau to prepare this report, is herewith submitted, marked L.

"XIV. An investigation by Dr. Elisha Harris, of New York, upon diphtheria, as it occurred in Northern Vermont, which is herewith submitted, marked M.

"XV. An investigation by Prof. Raphael Pumpelly, of the United States Geological Survey, upon the influence of various soils upon sanitation, especially with regard to drainage and methods of disposal of excreta. This has been but recently authorized."

The foregoing extract certainly indicates that the board has been active and comprehensive in the consideration of all subjects properly coming before it and in carefully planning investigations of importance, some of which will require several years to complete. The report next details what has been undertaken and accomplished in reference to quarantine regulations and the establishment of an international system of notification concerning the prevalence of infectious diseases.

The following extract concludes this part of the report:

"The great majority of quarantine establishments on our seaports are either improperly located or do not possess the facilities necessary for properly dealing with infected ships, many of them amounting to little more, practically, than inspecting and boarding stations. Owing to the epidemic of the previous summer, unusual care has been exercised by the inspecting and boarding officers at the various quarantine stations, and no satisfactory evidence has been presented to the board that any outbreak of contagious or infectious disease has occurred in this country from importation during the present year. That this is the case, however, must be attributed mainly to good fortune and the fact that no dangerously infected ship has arrived at certain quarantine stations.

"It is not considered by the board to be necessary or desirable that every seaport in the United States, nor even that the majority of ports, should possess a complete quarantine establishment, including boats, lighters, wharves, warehouses, hospitals and other buildings necessary to properly care for an infected ship and its cargo, passengers, and crew in such a manner that there shall be as little detention or interference with commerce as possible, since such establishments are expensive both to establish and to maintain, and at many ports no infected ship might arrive for several years.

"It has therefore been decided that, for the present at all events, such complete quarantine establishments are only needed at Boston, New York, Philadelphia, Baltimore, near the mouth of the Chesapeake Bay, Wilmington, Charleston, Savannah, near Brunswick, Ga., or Fernandina, Fla., near Ship Island, Miss., and at some point on the Texas coast.

"The station at the mouth of Chesapeake Bay would care for some, if not all, infected vessels bound for Baltimore, Annapolis, Washington, Fredericksburg, Richmond, Norfolk and Petersburg, and would deal also with infected vessels bound for northern ports, which in their distress and in an emergency may put into Hampton Roads. The station near Brunswick or Fernandina has not yet been positively decided on. If established, it would care for infected vessels destined for ports on the Atlantic coast between Savannah and Key West. The quarantine station at Ship Island would be devoted to the treatment of infected vessels destined for the Gulf ports between and including Pensacola and

New Orleans, which would include the smaller ports of the Gulf coast of Florida, Alabama, Mississippi and Louisiana. The total expense of establishing these three quarantine stations is estimated at $100,000, and as each one of them is for the protection of several States, it seems eminently proper and desirable that they should be equipped and maintained by the United States.

"In addition to this there should be expended at other ports about $65,000. This work should be done at once in order to secure during this winter, as far as possible, satisfactory quarantine arrangements on the South Atlantic and Gulf coasts, to secure the prevention of the introduction of yellow fever through any of these ports. It is the opinion of the board that if, during the present winter, at all points where the fever has prevailed during the past summer, care be taken to obtain thorough ventilation and exposure to cold of all houses and inclosed spaces, and of all bedding, clothing, etc., and if local sanitation be vigorously and properly carried out, there will be little danger of an epidemic of yellow fever next year from causes now existing in the country. The board is using all its influence to induce local authorities to insure this cleansing, ventilation, etc., and has reason to believe that much will be accomplished before warm weather again sets in. Under these circumstances, the board is of the opinion that it is its duty to use the means at its command to have the maritime quarantine stations of the country placed in proper condition at once, in order that they may be ready to meet any emergency not later than the first of May; and it therefore proposes to go on and carry out the plans above suggested without delay.

"With regard to inland quarantines and quarantine stations, much must of course depend upon the existence of contagious or infectious disease, requiring their establishment. Irrespective of this, however, it is believed by the board that inspecting stations for steamboats should be established on the Mississippi river, at which all boats should be examined by competent inspectors, and certificates as to the sanitary condition of the boat given. This inspection should be carried on throughout the summer at all events, irrespective of the existence of yellow fever or other contagious or infectious disease in New Orleans or other ports upon the river, and it should apply to boats going south as well as to those going north. That such an inspection, under the auspices of this board, would be acceptable to owners of steamboat lines on the Mississippi, the board has satisfactory evidence.

"Inspecting stations for this purpose should be located at New Orleans, Vicksburg, just below Memphis, and at Cairo. At each of these stations inspecting officers should be provided with a steam-launch so arranged that in most cases the inspection could be made with little or no hinderance, or but slight delay, to the boat. At Vicksburg, Memphis, and Cairo, the stations should be so located that arrangements can be made on shore, by means of tents, etc., to deal with an infected boat should occasion present. In addition to these stations and the boats, tents, and furniture connected with them, there should be provided for the use of the board on the Mississippi river a small and fast steamboat to be used as a patrol boat, more especially for visiting places on the river reported as infected, but which are remote from railroad communication. It is often extremely difficult to obtain access to such places within any reasonable space of time, since none of the steamboats plying on the river will land at them for fear of being quarantined at other points, and there is usually a non-intercourse, "shot-gun" quarantine established around them from the land side. The result is the rapid spread of the fever, great suffering, and ultimate danger to the surrounding country. Had the board had such a boat as above indicated at its command during the past summer it is probable that relief might have been promptly afforded to Concordia and vicinity, and that much suffering and loss of life might have been prevented. The total cost for establishing these inspecting stations and for the purchase of a small steamboat which shall be capable of making about fifteen miles an hour against the current and carrying three or four tons of freight in the shape of medical supplies, etc., is estimated at $35,000, and the board proposes to proceed at once to take the necessary steps to carry out this recommendation for the same reason as that given for acting at once with regard to the maritime quarantines, viz, that they may be ready for active operations not later than the first of May."

A brief history of the origin and progress of the yellow fever in Memphis and New Orleans in 1879 is given; following which are the rules and regulations governing the board in regard to the rendering of aid to State and local boards of health, which have already been published in this journal. The report closes with a financial estimate of the amount of money that will be needed for carrying on its operations during the next fiscal year. The total expenditures of the board to the present date are $154,002.42. The amount needed for the next six months is estimated to be $284,330 and for the twelve months following, carrying the time to June 30, 1881, $202,050; making the total asked for to meet the wants of the board for the next eighteen months, $486,390.

Not only from this annual report, but from all we have learned of the doings of the national board thus far, we are satisfied that its members have devoted all the time and attention to the duties assigned them that could have been reasonably expected; and have accomplished as much as was possible to accomplish in the brief time which has elapsed since their appointment.

In regard to the abstract question, whether the permanent establishment of a national board of health is desirable or necessary, we would say that from principle we are opposed to that tendency which is everywhere apparent, to look to government for doing those things that the people are capable of doing for themselves; and especially are we opposed to the multiplication of governmental departments, bureaus, boards, etc. For, the more complicated is made the machinery of governments, the greater is the expense in proportion to the work accomplished. As all regulations in relation to the gathering of vital statistics and the carrying on of local sanitary improvements must emanate from the State and municipal governments, leaving the general government with proper jurisdiction over the sanitary matters connected with the army, navy, and commercial intercourse between the States and with foreign countries only, it has seemed to us that the already efficiently organized medical departments of the army, navy and Marine Hospital service, afforded all necessary channels for the operations of the general government, while the American Public Health Association constitutes a very proper head and bond of union for the State and local sanitary and health organizations. D.

